# Dermatological evaluation in dogs with atopic dermatitis treated with full-spectrum high cannabidiol oil: a pre study part 1

**DOI:** 10.3389/fvets.2023.1285384

**Published:** 2023-10-31

**Authors:** Carollina Mariga, Ana Lúcia Souza Silva Mateus, Ângela Isabel dos Santos Dullius, Ana Paula da Silva, Mariana Martins Flores, André Vasconcelos Soares, Erik Amazonas, Saulo Tadeu Lemos Pinto Filho

**Affiliations:** ^1^Programa de Pós-Graduação em Medicina Veterinária pela Universidade Federal de Santa Maria (UFSM), Santa Maria, Rio Grande do Sul, Brazil; ^2^Departamento de Estatística na UFSM, Santa Maria, Rio Grande do Sul, Brazil; ^3^Departamento de Clínica Médica no Hospital Veterinário Universitário da UFSM, Santa Maria, Rio Grande do Sul, Brazil; ^4^Laboratório de Patologia Veterinária (LPV-UFSM), Santa Maria, Rio Grande do Sul, Brazil; ^5^Departamento de Clínica de Pequenos Animais, Santa Maria, Rio Grande do Sul, Brazil; ^6^Departamento de biociências e saúde única da Universidade Federal de Santa Catarina (UFSC), Curitibanos, Santa Catarina, Brazil

**Keywords:** canine atopic dermatitis, cannabidiol, cannabis, histopathology, mast cell count, veterinary dermatology

## Abstract

**Introduction:**

Dermatological consultations represent a great part of the small animal medical clinic routine. Canine atopic dermatitis (CAD) is a common skin disease that affects a significant amount of dogs, making it a relevant consideration in clinical practice. The role of the endocannabinoid system on skin homeostasis has been described and its deregulation contributes to dermatopathies. Its function in specialized skin cells reveals an expressive therapeutic potential. Due to the difficulties and the growing scientific evidence of the therapeutic benefits of cannabis on animals, this work aimed to evaluate the anti-inflammatory effects of cannabis-derived oil in the treatment of CAD.

**Methods:**

Fourteen canines diagnosed with CAD were divided into two groups: T: full spectrum high cannabidiol (CBD) cannabis oil, 2,5 mg/kg; and C: control group (treated with olive oil alone). The effectiveness was evaluated based on the degree of pruritus, dermatological evaluation (CADESI-4) and histopathological evaluation of the skin including mast cell count.

**Results:**

Despite the theoretical basis, there were no significant results obtained between the compared treatments.

**Discussion:**

Thus, it can be concluded that although full spectrum high cannabinoids therapy presents a promising approach to immunological diseases, further research is required in order to establish the actual effective cannabinoid ratio within the myriad possible combinations and for multi-target therapy of CAD.

## Introduction

Canine atopic dermatitis (DAC) is a disorder resulting in chronic inflammation and pruritus (1, 2). DAC is multifactorial and its pathogenicity is not well elucidated. It is known to be genetic predisposition with immunological alterations leading to defective cutaneous barriers and hyperinflation of the skin (3). The syndrome begins with sensitivity to environmental allergens, mainly house dust mites, which penetrate the skin and stimulate the recruitment of inflammatory cells and IgE-mediated mast cell degranulation (2, 4). The management of this disease requires a multimodal therapy aimed at improving the skin barrier, immunomodulation, and prevention of allergies (5).

According to Tóth et al. (6), the endocannabinoid system plays a key role in skin homeostasis, barrier formation, and regeneration. Disruptions to this system can lead to diseases and disorders, such as atopic dermatitis. The endocannabinoid system is present in immune cells and skin-specific cells such as keratinocytes, fibroblasts, melanocytes, and sebocytes, making them all potential therapeutic targets (7). Current literature suggests that the effects of cannabinoids and cannabinoid-related receptors on specialized skin cells modulate inflammation and offer a novel approach to treating atopic dermatitis by regulating different mechanisms of the disease (8).

In light of the strong relationship between the endocannabinoid system and skin homeostasis, the objective was to evaluate the effectiveness of full-spectrum cannabis oil rich in CBD in dogs with atopic dermatitis through dermatological assessment, using CADESI-4 and pruritus degree, as well as histopathological analysis with mast cell counting in three regions affected by atopic dermatitis.

## Materials and methods

### Ethics committee

This study was approved by the Ethics Committee for Animal Use and Experimentation of the Federal University of Santa Maria (CEUA/UFSM) (number 8656301121 - ID 003662) and was conducted in accordance with the ethical principles of the National Council for Animal Experimentation Control (CONCEA).

### Animal selection

Canine subjects with a diagnosis of atopic dermatitis (AD), with prior exclusion of food allergy and flea bite dermatitis, were selected according to the criteria of Favrot.

The inclusion criteria were: AD diagnosis; Absence of concurrent diseases; No systemic treatment within the past 30 days; The exclusion criteria were: Lack of regular flea control; Presence of dental calculus; Moderate-to-severe gingivitis; Any other concurrent disease. No maximum or minimum CADESI values were stipulated for selection or exclusion. Additionally, there was no standardization in the use of shampoo for topical treatment or type of diet used, in order to mimic clinical routine.

The fourteen canines (Table 1) were divided into two groups: T: the treatment group, treated with cannabis oil, and C: the control group, treated with olive oil (diluent of the product used in the treatment group with maximum acidity of 0,4%). The cannabis oil used contained a full spectrum with a higher concentration of cannabidiol (CBD) at 1,500 mg, in a ratio of 21:1 for CBD:THC (AMA+ME^®^). The treatment involved administering 2.5 mg/kg, twice a day for 60 days. Assessments were conducted before (T0) and after (T60) the treatment. The frequency of baths, shampoos, and diets already in use, whether therapeutic or not, was maintained. It was also recommended to withhold any type of treat from the animal.

**Table 1:** Animal demographics.

**Group**	**Sex**	**Age**	**Race**	**Age of disease**	**Previous treatment**
**Control**					
Animal 1	Male	12 years	Shih-tzu	6 months	Corticosteroid
Animal 2	Female	1 year	Lhasa apso	9 months	Corticosteroid
Animal 3	Female	5 years	Shih-tzu	1 year	Oclacitinib
Animal 4	Female	1 year	Dachshund	10 months	Corticosteroid, oclacitinib
Animal 5	Female	11 years	Shih-tzu	1 year	Corticosteroid, oclacitinib, caninized monoclonal antibody
Animal 6	Male	7 years	Shih-tzu	3 years	Corticosteroid
Animal 7	Male	6 years	Dachshund	3 years	Corticosteroid
**CBD**					
Animal 1	Female	8 years	Shih-tzu	6 months	Corticosteroid
Animal 2	Female	10 years	Shih-tzu	3 years	Corticosteroid, oclacitinib, caninized monoclonal antibody
Animal 3	Male	6 years	Golden Retriever	1 year	Corticosteroid
Animal 4	Female	7 years	Shih-tzu	3 years	Corticosteroid
Animal 5	Female	8 years	Lhasa apso	2 years	Corticosteroid, oclacitinib
Animal 6	Male	9 years	Shih-tzu	1 year	Corticosteroid
Animal 7	Male	11 years	Shih-tzu	3 years	Corticosteroid, oclacitinib

### Dermatological evaluation

The animal was assessed by a dermatologist using the CADESI-4 score, and according to the owner, the degree of pruritus was evaluated using the scale adapted. Both the dermatologist and the owner were blinded to the treatment.

### Histopathology and mast cells count

For skin biopsy, intravenous sedation was performed using 3 μg/kg fentanyl and 4 μg/kg of dexmedetomidine (Dexdomitor^®^, Zoetis). The biopsy was taken from the interdigital, axillary, and inguinal regions with the aid of a scalpel or punch. The tissue samples were preserved in 10% formalin for subsequent histopathological evaluation and mast cell counting using special toluidine blue staining under a 400× objective. Suturing was done using 3-0 Sultan nylon sutures.

The histopathological evaluation criteria included (a) Orthokeratotic hyperkeratosis, (b) acanthosis, and (c) perivascular and periadnexal lymphoplasmacytic dermal inflammation. These criteria were subsequently classified into four grades (absent, mild, moderate, and severe) for the interdigital, axillary, and inguinal regions.

### Statistics analysis

Descriptive statistics were performed for qualitative variables, including calculating frequencies (both absolute and relative), and for quantitative variables, measures of central tendency and variability were calculated. The Shapiro-Wilk test was conducted to test the normality of the data. For comparisons between two groups, the Student's *t*-test (independent groups) or paired *t*-test (dependent groups) were used when the data did not follow a normal distribution. The Mann-Whitney *U* test (independent groups) or Wilcoxon signed-rank test (dependent groups) were applied in case of non-normality. The Chi-Square test was used for the association between qualitative variables. Statistical Package for Social Sciences (SPSS) version 17.0 software was used for the analyses, and the significance level was set at 5%.

## Results

### Dermatological evaluation

**Figure 1:**
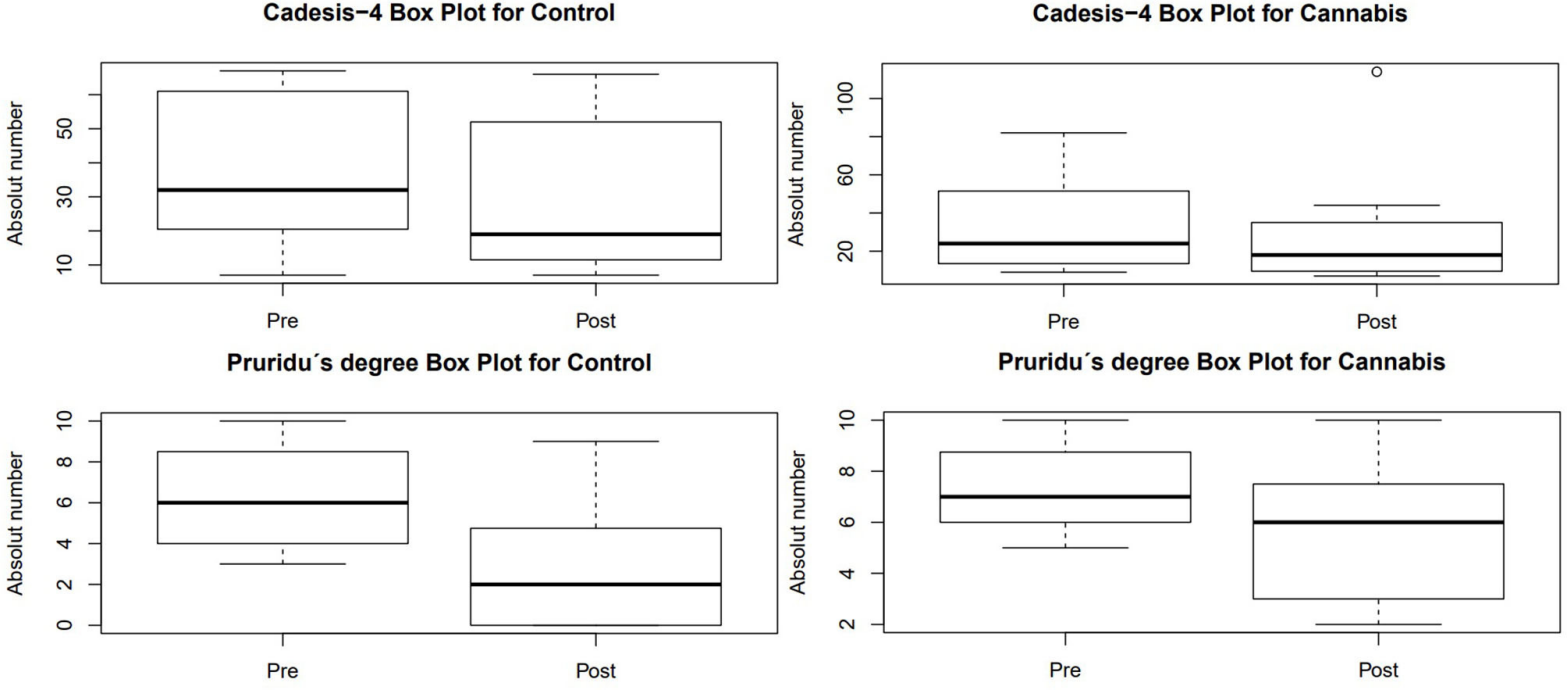
Box plot obtained in the evaluations of CADESI-4 and degree of pruritus in the pre and pros-treatment groups.

In the CADESI-4 evaluation (Table 2), there was a difference in pre- and post-treatment for group C (*p* = 0,042), but there was no difference within group T (*p* = 0,398) or between the treatments performed (*p* = 0.654). The limit values obtained from this evaluation are shown in Figure 1.

**Table 2:** Pre- and post-treatment CADESI-04, itching degree (PVAS), and mast cell evaluation in dogs with atopic dermatitis.

**Group**	**Animal**	**CADESI-04**		**Itching degree**		**Mast cells**					
						**Armpit**		**Interdigit**		**Groin**	
		**Pre**	**Post**	**Pre**	**Post**	**Pre**	**Post**	**Pre**	**Post**	**Pre**	**Post**
Control	1	67	44	7	2	62	104	334	172	186	146
	2	14	18	9	8	84	102	296	134	76	46
	3	36	26	5	7	88	94	196	293	105	120
	4	13	7	5	6	138	45	60	143	34	43
	5	82	114	10	10	96	78	226	136	122	165
	6	24	12	8.5	2	66	90	6	168	80	67
	7	9	7	7	4	96	52	110	124	50	82
CBD	1	32	19	3	0	110	156	202	350	102	360
	2	18	7	9	4.5	102	176	360	320	146	90
	3	7	11	8	9	440	101	248	114	34	51
	4	57	49	4	5	228	232	404	260	167	230
	5	23	12	6	0	115	152	304	98	112	178
	6	65	55	4	0	155	148	404	203	194	161
	7	67	66	10	2	103	84	242	208	205	110

Pre, pre-treatment; Post, post-treatment.

Regarding the degree of pruritus (Table 2), assessed according to the tutor's evaluation, there was no difference the tested treatments (*p* = 0.396), as well as in the pre- and post-treatment within group T (*p* = 0.186), but there was a significant decrease in group C (*p* = 0.039) (Figure 1).

### Histopathology and mast cells count

In the axillary region (Table 3), group T showed no changes in five animals (71.4%) in the evaluation of orthokeratotic hyperkeratosis, but it revealed a worsening of the lesion grade in two animals, with one previously classified as absent but now showing moderate lesions. As for the presence of acanthosis, five animals (71.4%) also showed no alteration, while two presented a worsening of the clinical condition. Perivascular inflammation remained in three animals (42.9%), and two showed an improvement in the evaluation grade, while two showed worsening. On the other hand, group C had four dogs (57.1%) with the same grade of Orthokeratotic hyperkeratosis, two with worsening, and one with a significant improvement, progressing from a moderate grade to absent. Concerning acanthosis, four patients (57.1%) maintained the same grade, while two (28.6%) showed improvement, and the only one that worsened progressed from absent to moderate. Perivascular inflammation remained in only one animal, while three (42.9%) showed worsening, and two (28.6%) showed improvement, with one representative experiencing a decrease in the grade from severe to mild.

**Table 3:** Histopathological findings in dogs with atopic dermatitis treated with high-CBD oil.

**Group**	**Animal**	**Orthokeratotic hyperkeratosis**						**Acanthosis**						**Perivascular and periadnexal dermal lymphoplasmacytic inflammation**					
	**Armpit**		**Groin**		**Interdigit**		**Armpit**		**Groin**		**Interdigit**		**Armpit**		**Groin**		**Interdigit**	
	**Pre**	**Post**	**Pre**	**Post**	**Pre**	**Post**	**Pre**	**Post**	**Pre**	**Post**	**Pre**	**Post**	**Pre**	**Post**	**Pre**	**Post**	**Pre**	**Post**
Control	1	G	G	G	G	Mo	G	Mo	Mo	Mo	Mo	Mo	Mo	G	Mo	Mo	Mo	Mo	Mo
	2	A	Mo	G	G	G	Mo	A	G	A	G	G	G	A	G	A	G	Mo	Mo
	3	A	A	Mo	A	Mo	G	A	G	G	Mo	Mo	Ma	G	G	G	G	Mo	Ma
	4	Mo	Mo	Mo	Mo	Ma	Mo	A	A	A	A	A	G	G	G	G	G	G	Mo
	5	G	G	G	G	Mo	G	G	G	Mo	Mo	Mo	Mo	Mo	G	Mo	G	Mo	Mo
	6	G	Mo	G	G	G	G	A	A	A	G	Mo	Mo	G	G	Mo	G	Mo	Mo
	7	Mo	Mo	G	Mo	A	Ma	A	A	A	G	A	A	G	A	A	G	G	G
CBD	1	G	Mo	G	G	Ma	A	A	Mo	G	Mo	G	G	G	Mo	Ma	Mo	Mo	Mo
	2	G	G	G	A	Mo	Ma	A	A	A	A	G	G	G	G	G	G	Mo	Mo
	3	Mo	A	G	G	G	A	G	A	A	A	Mo	A	Ma	G	G	G	G	G
	4	G	G	G	G	Mo	G	Ma	Ma	Mo	Ma	Mo	Mo	Ma	Mo	Mo	Ma	Ma	Mo
	5	Mo	Mo	Mo	Mo	G	Mo	A	A	A	G	Mo	G	G	Mo	G	Mo	Mo	G
	6	G	G	G	Mo	G	Mo	Ma	Ma	Ma	G	G	Ma	Ma	Mo	Ma	Mo	Mo	Mo
	7	Mo	Ma	Mo	G	G	G	Ma	Mo	Mo	G	G	Mo	Mo	Ma	Mo	G	Mo	Mo

Pre, pre-treatment; Post, post-treatment; A, Absent; G, Gentle; Mo, Moderate; Ma, Marked.

In the interdigital region (Table 3), group T showed a 57.1% improvement in Orthokeratotic hyperkeratosis lesions, with one remaining at the same level and two experiencing worsening, including one from absent to severe. As for the presence of acanthosis in the same group, 71.4% remained at the same grade, while two showed worsening. Perivascular inflammation had the same proportion as acanthosis, but without a significant change in the grade of the lesion. In group C, there was an equal proportion (42.9%) of animals with worsening and improvement in the grade of Orthokeratotic hyperkeratosis, except for one animal that progressed from severe to absent, and only one maintained the same grade. Regarding acanthosis in this group, three (42.9%) maintained the same grade of the lesion, and the same proportion (28.6%) showed worsening and improvement, progressing from mild to severe and moderate to absent, respectively. Perivascular inflammation in the control group revealed 71.4% maintaining the grade and two with and improvement in the grades previously presented.

In the inguinal region (Table 3), Orthokeratotic hyperkeratosis in group T remained in 57.1% of the animals, with a worsening of grade in only one and improvement in two patients, with one progressing from moderate to absent. As for the evaluation of acanthosis, maintenance was observed in 42.9% of the animals, while 57.1% showed worsening. Concerning perivascular inflammation, three animals maintained the same grade, and two (28.6%) showed improvement and worsening. Group C presented 57.1% maintenance in Orthokeratotic hyperkeratosis lesions, two showed improvement, and two worsened in grade. As for the evaluation of acanthosis, three (42.9%) showed worsening, two remained at the same grade, and two improved, with one progressing from severe to mild. Regarding perivascular inflammation, 42.9% showed improvement, and 28.6% showed worsening and maintenance of the grade.

**Figure 2:**
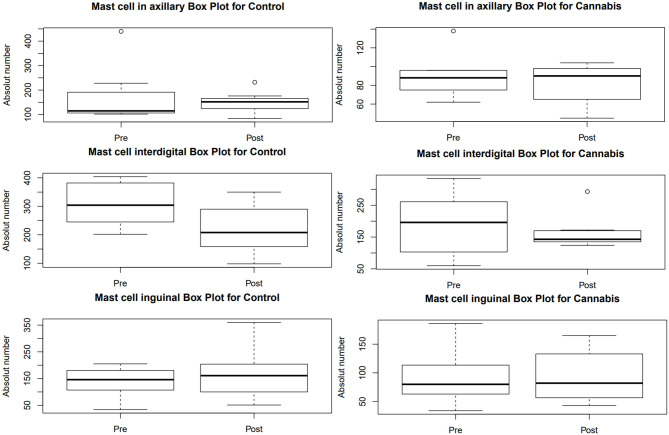
Box plot obtained in the mast cell count performed on biopsies with special toluidine blue staining at 400× magnification in dogs with AD. Pre, pre-treatment; Post, post-treatment.

The mast cell count (Table 2) did not show statistical difference between pre- and post-treatment within and between the groups (Figure 2).

## Discussion

### Dermatological evaluation

The onset of clinical signs of canine atopic dermatitis (CAD) is characterized by alesional pruritus, represented by excessive scratching, biting, or licking. The primary lesions are erythema and papules, which may lead to secondary lesions due to self-trauma, resulting in chronic inflammation and secondary infections, represented by excoriations, alopecia, lichenification, hyperpigmentation, crusting, and seborrhea (1). Luz-Veiga et al. (9) suggest that phytocannabinoids CBD and CBG have promising antimicrobial effects when topically Applied, without altering the skin's microbiota, which could become an ally in the treatment of dermatopathies. Campora et al. (10) detected increased immunoreactivity of CB1 and CB2 receptors in various cell types in the epidermis and dermis of dogs diagnosed with atopic dermatitis, including mast cells, fibroblast, and endothelial cells.

**Figure 3:**
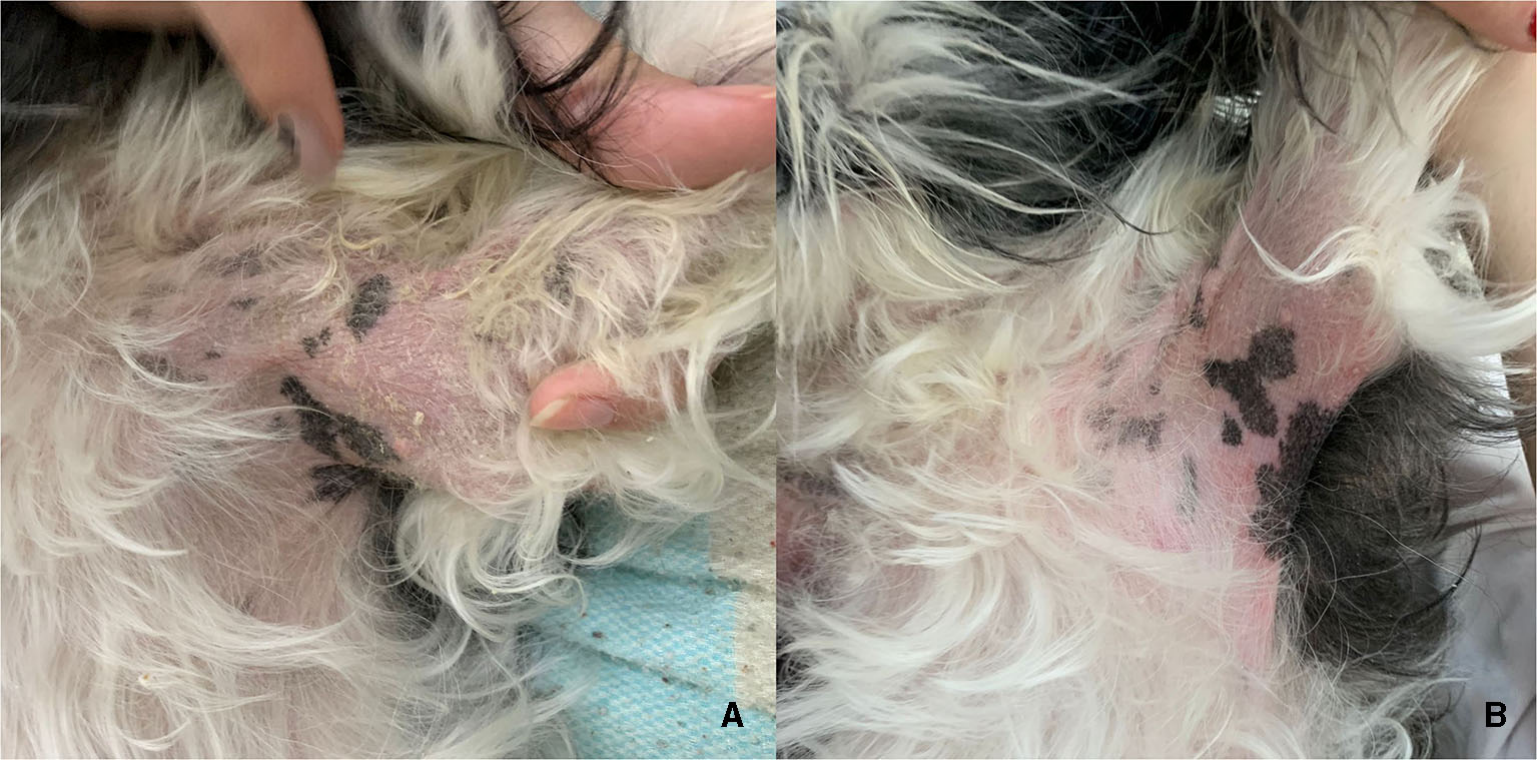
Axilla of a dog from the group treated with CBD-rich cannabis oil, before **(A)** and after **(B)** 60 days of treatment. Notice the reduction of erythema and absence of scaling in **(B)**.

In a treatment with 2 mg/kg of CBD and CBDA for 4 weeks conducted by Loewinger et al. (11) in atopic dogs and evaluated using CADESI-4 and pruritus scores, no statistical improvement was observed. This study corroborates with the current study, which also did not find significance in these analyses, even when extended to 6 weeks of treatment. It is worth nothing that the treatment duration may be considered short to observe significant improvement in the skin. Additionally, we emphasize that there were no specific maximum and minimum limits for CADESI-4 evaluation for inclusion and/or exclusion in this study, as stipulated by Loewinger et al. (11). However, Mogi et al. (12) revealed clinical improvement in both CADESI-4 and pruritus scores in atopic dogs treated with THC-free CBD oil twice daily for 8weeks. However, this study did not include a control group for comparison of the obtained data. Despite the lack of significance in laboratory analyses, Figure 3 shows clinical improvement presented by a dog in the cannabis-treated group, which none of the dogs in the control group showed.

A study conducted by de Santiago et al. (13) revealed that a diet with high concentrations of antioxidants, polyphenols, docosahexaenoic omega-3 fatty acids, and eicosapentaenoic acid improves skin health, reduces inflammation, and enhances clinical signs of CAD, as assessed by CADESI-4 and pruritus scores. The lack of significance between the treatment groups and the significance observed between the pre- and post-treatment within the control group can be attributed, primarily, to the non-inert diluent, olive oil, used in the control group. More than 50 different phenols have been identified in olive oil wastewater, and they have been associated with antioxidant, anti-inflammatory, and antimicrobial properties (14, 15). The current literature diverges on the efficacy of olive oil in atopic dermatitis, with some authors justifying positive results when administered concomitantly with other substances (16). There are no references found on the effects of oral administration of olive oil for dermatopathies, especially for canine atopic dermatitis. Additionally, it is essential to consider the various pruritogenic mechanisms involved, including hyperinnervation of CAD lesions, pro-inflammatory molecules, keratinocytes, monocytes, cutaneous nerve fibers, and the involvement of the central and peripheral nervous systems (17, 18). Although the complete understanding of pruritus is still lacking, it had been shown that TRPV1 activation minimizes itching induced by dust mite allergens in mice with atopic dermatitis (19), the main allergen in dogs of this region reported by Pereira et al. (4).

The results obtained according to the responses of the tutors regarding the pruritus grade disagreed with the dermatologist's evaluation. There are a few studies on the placebo effect in dogs, but there are some theories that do not involve a real placebo effect. (A) Placebo effect of the caregiver or by proxy: this means that due to the investment made, the caregiver believes that they will get a return. As a result, the tutor is highly susceptible to this type of control and may perceive improvements even when they do not exist. A study conducted by Conzemius and Evans (20) reported a prevalence of 39.7% and 44.8% of proxy control effects in dogs with lameness as evaluated by tutors and veterinarians, respectively. (B) Regression to the mean: this refers to a real improvement in the animal, but it occurs as a natural course of regression. This is associated with chronic diseases that fluctuate in severity naturally. A study conducted by Muñana et al. (21) concluded that 79% of dogs that received control for epilepsy showed improvement in seizure frequency. Both types of effects emphasize the importance of having a control group in scientific research in veterinary medicine to ensure that the evidence obtained is not poor and to accurately evaluate the success or failure of the intervention.

Given the chronicity, difficulty in controlling clinical signs, and the laborious maintenance of canine atopic dermatitis, the placebo effect concerning the tutor's evaluation can be a reasonable justification, even if not well-supported by the literature. There are reports of improvement according to some tutors, but it is not observed in all cases, which can be related to the placebo effect described above. In other words, there may have been a clinical improvement, but without statistical significance. Recalling the discussion in the “dermatological evaluation” section, it was possible to justify an improvement in the control group because it was not treated with an inert substance, thereby approximating the possible differences between the cannabis-treated group and the control group.

It is believed that the main limitation regarding the pruritus scale was the lack of prior information about the pruritus grade. As a result, tutors would confirm a certain level of pruritus that was often higher than the confirmed previous grade, subsequently reporting that the pruritus had indeed decreased. This discrepancy in reporting could be attributed to the absence of initial information and may have influenced the perceived improvement in pruritus.

### Histopathology and mast cells count

The presence of mast cells is directly proportional to the severity of the clinical condition, pruritus pathogenesis, and disease progression (22). The results obtained from the tutor's assessment of pruritus grade are consistent with the results regarding the number of mast cells. Despite the mention of the possibility of the placebo effect in tutors, the number of mast cells did not show statistical difference in dogs with atopic dermatitis treated with cannabis. Nam et al. (23) reported a reduction in activation and degranulation of mast cells, as well as decreased recruitment of these cells and local inflammation in atopic dermatitis through CB1 receptors. The antipruritic action is also attributed to mast cells, primarily related to the endocannabinoid PEA (24). Additionally, Mogi et al. (12) emphasized the importance of adding phytocannabinoid supplementation early in the disease course, as using it as a single agent in refractory or severe cases does not show significant clinical improvement. The same authors also advocate combining phytocannabinoids with conventional drugs to potentially reduce dosages, financial costs, and enhance the overall efficacy of the treatment plan. It is essential to highlight that the anti-inflammatory mechanisms are not yet fully understood (25).

The histopathological lesions of CAD reveal an inflammatory pattern characterized by chronicity, perivascular dermatitis, hyperplasia, and spongiosis (26). Scott (27) reported the presence of epidermal hyperplasia, orthokeratotic or parakeratotic hyperkeratosis, hypergranulosis, spongiosis, melanosis, and leukocytic exocytosis. This same author also mentioned dermal changes such as congestion, vasodilation, and angiocentric inflammation with predominantly mononuclear and neutrophilic infiltrated. Campora et al. (10) revealed hyperplastic epidermis and focal hyperkeratosis in five dogs with CAD. In a detailed study, Chiocchetti et al. (8) described the presence of moderate to severe hyperkeratosis and acanthosis, with focal to diffuse distribution in eight dogs with CAD. They also reported superficial and interstitial perivascular inflammatory infiltrates, consisting of cells such as lymphocytes, histiocytes, mast cells, plasma cells, and some eosinophils. In four of these animals, predominantly neutrophils were present. The latter authors did not disclose the biopsy site or whether any treatment was used. Chiocchetti et al. (8) aimed to investigate the expression of CB2, GPR55, TRPV1, and TRPA1 receptors in skin cells of dogs with atopic dermatitis. The authors concluded that these receptors are highly expressed in infiltrative inflammation in dogs with atopic dermatitis and that cannabis has a considerable theoretical basis as a potential therapeutic option for this disease, alleviating pruritus and inflammation. The findings of the present study, which provide a more detailed description of the histopathological changes in the most affected regions of dogs with atopy, contradict the literature that supports the therapeutic potential. In other words, no improvement in the evaluation criteria specified in this study was observed.

The absence of significant findings in the different evaluations conducted in this study can be mainly justified by the fact that the animals had a dermatopathy, and these changes are primarily related to the dose used. In the present study, the body condition of each patient was not taken into consideration, whereas in the literature, there is evidence of phytocannabinoid deposition in adipose tissue due to their liposolubility. Studies that accounted for this factor revealed an increase of 20% in the dose for obese animals to compensate for this characteristic (28).

Limitations of this study include: small number and uniformized animals; short-term therapy; and possible influence of olive oil. However, despite the absence of clinical improvement in this study, cannabinoids are a promising option to ameliorate the pathophysiology of this disease.

## Conclusion

Despite the absence of significance in the dermatological evaluations for the canine atopic dermatitis, it is worth noting the individuality of each animal concerning the dosage used, as a very positive result was obtained in the cannabis group. This study reveals that the full-spectrum cannabis oil rich in CBD at a dosage of 2.5 mg/kg does not show therapeutic advantage when compared to olive oil. This is mainly due to the complexity of controlling this disease, which demands a multimodal therapy. Further clinical research involving this topic is recommended to either confirm or definitively rule out potential therapeutic means to aid in controlling this dermatopathy that greatly affects the quality of life.

## Data availability statement

The raw data supporting the conclusions of this article will be made available by the authors, without undue reservation.

## Ethics statement

The animal studies were approved by Ethics Committee for Animal Use and Experimentation of the Federal University of Santa Maria (CEUA/UFSM) (protocol number 8656301121, identification number 003662). The studies were conducted in accordance with the local legislation and institutional requirements. Written informed consent was obtained from the owners for the participation of their animals in this study.

## Author contributions

CM: Conceptualization, Data curation, Formal analysis, Investigation, Methodology, Writing—original draft, Writing—review & editing. ALS: Conceptualization, Data curation, Formal analysis, Methodology, Software, Validation, Writing—original draft, Writing—review & editing. ÂIS: Data curation, Formal analysis, Writing—review & editing. APS: Writing—review & editing, Formal analysis. MM: Investigation, Methodology, Writing—review & editing. AVS: Writing—review & editing, Methodology, Supervision. EA: Conceptualization, Resources, Writing—review & editing, Funding acquisition. ST: Conceptualization, Formal analysis, Funding acquisition, Investigation, Methodology, Project administration, Resources, Supervision, Validation, Writing—review & editing.
